# Randomized Controlled Trial Study of the Impact of a Spiritual Intervention on Hope and Spiritual Well-Being of Persons with Cancer

**DOI:** 10.17533/udea.iee.v39n3e08

**Published:** 2021-11-08

**Authors:** Ardashir Afrasiabifar, Asadollah Mosavi, Abolfazl Taghipour Jahromi, Nazafarin Hosseini

**Affiliations:** 1 Professor, Ph.D. School of Nursing, Yasuj University of Medical Sciences, Yasuj, Iran Email: afrasiabifar.ardashir@yums.ac.ir Yasuj University of Medical Sciences School of Nursing Yasuj University of Medical Sciences Yasuj Iran afrasiabifar.ardashir@yums.ac.ir; 2 Nurse Instructor, M.Sc. School of Nursing, Yasuj University of Medical Sciences, Yasuj, Iran Email: mosaviasadolah@yahoo.com. Corresponding author. Yasuj University of Medical Sciences School of Nursing Yasuj University of Medical Sciences Yasuj Iran mosaviasadolah@yahoo.com; 3 Nurse, M.Sc. School of Nursing, Yasuj University of Medical Sciences, Yasuj, Iran Email: ataghpour38@gmail.com Yasuj University of Medical Sciences School of Nursing Yasuj University of Medical Sciences Yasuj Iran ataghpour38@gmail.com; 4 Associate Professor, Ph.D. School of Nursing, Yasuj University of Medical Sciences, Yasuj, Iran Email: hosseinichenar@yahoo.com Yasuj University of Medical Sciences School of Nursing Yasuj University of Medical Sciences Yasuj Iran hosseinichenar@yahoo.com

**Keywords:** patiens, neoplasms, hope, spirituality., pacientes, neoplasias, esperanza, espiritualidad**.**, patients, neoplasias, esperança, espiritualidade.

## Abstract

**Objective:**

To determine the impact of spiritual intervention on hope and spiritual well-being of persons with cancer.

**Methods:**

Randomized controlled trial in which 74 patients with cancer referring to a chemotherapy ward of Shahid Rajaie Hospital in Yasuj city, Iran, were participated. The eligible patients were randomly assigned to either intervention or control group. Spiritual-based intervention was performed based on the protocol in four main fields namely; religious, existence, emotional and social over 5 sessions before chemotherapy. The participants in the control group had received usual cares. Data were collected using Snyder's Hope Scale and Ellison's Scale Spiritual Well-Being Scale on a week before and after intervention.

**Results:**

The total mean scores of the scales of hope and spiritual well-being in both groups did not present statistical differences in the pre-intervention assessment. In contrast, at the post assessment, significant differences (*p*<0.001) were found in the mean scores between the intervention and control groups on the hope scale (60.9 versus 39.8) and on the spiritual well-being scale (94.3 versus 71.6).

**Conclusion:**

Spiritual intervention could promote hope and spiritual well-being of persons with cancer.

## Resumen

**Objetivo:**

Determinar el impacto de una intervención espiritual en la esperanza y el bienestar espiritual de las personas con cáncer.

**Métodos:**

Ensayo controlado aleatorio en el que participaron 74 pacientes con cáncer que acudieron a una sala de quimioterapia del Hospital Shahid Rajaie de la ciudad de Yasuj (Irán). Los pacientes elegibles se asignaron aleatoriamente al grupo de intervención o al de control. Durante 5 sesiones, y antes de la quimioterapia, se llevó a cabo una intervención espiritual basada en un protocolo con cuatro campos principales: religioso, existencia, emocional y social. Los participantes en el grupo control recibieron el cuidado usual. Los datos se recogieron mediante la aplicación de la escala de esperanza de Snyder, una semana antes y una semana después de la intervención, y de la escala de bienestar espiritual de Ellison.

**Resultados:**

Las puntuaciones medias de las escalas de esperanza y bienestar espiritual en ambos grupos no presentaron diferencias estadísticas en la evaluación pre-intervención. En cambio, en la evaluación posterior, se encontraron diferencias significativas (*p*<0.001) en las puntaciones medias entre los grupos de intervención y de control en la escala de esperanza (60.9 frente a 39.8) y en la escala de bienestar espiritual (94.3 frente a 71.6).

**Conclusión:**

La intervención espiritual podría promover la esperanza y el bienestar espiritual de las personas con cáncer.

## Resumo

**Objetivo:**

.Determinar o impacto de uma intervenção espiritual na esperança e no bem-estar espiritual das pessoas com câncer.

**Métodos:**

Ensaio controlado aleatório envolvendo 74 pacientes com câncer que frequentaram uma sala de quimioterapia no Hospital Shahid Rajaie na cidade de Yasuj (Irã). Os pacientes elegíveis foram aleatoriamente designados para o grupo de intervenção ou controle. Durante 5 sessões, e antes da quimioterapia, foi realizada uma intervenção espiritual baseada em um protocolo com quatro campos principais: religioso, existencial, emocional e social. Os participantes do grupo controle receberam os cuidados habituais. Os dados foram coletados por meio da aplicação da Escala de Esperança de Snyder e da Escala de Bem-Estar Espiritual de Ellison uma semana antes e uma semana após a intervenção.

**Resultados:**

Os escores médios das escalas de esperança e bem-estar espiritual em ambos os grupos não apresentaram diferenças estatísticas na avaliação pré-intervenção. Por outro lado, na avaliação subsequente, foram encontradas diferenças significativas (*p* <0.001) nas pontuações médias entre os grupos intervenção e controle na escala de esperança (60.9 vs. 39.8) e na escala de bem-estar espiritual (94.3 vs. 71.6).

**Conclusão:**

A intervenção espiritual pode promover esperança e bem-estar espiritual para pessoas com câncer.

## Introduction

The diagnosis of cancer is considered as a crisis by patients and their families in the most times.(1) In addition, the effect of cancer on patients’ physical and psychosocial health,(2) cancer may reduce their life expectancy due to re-hospitalization and complications of treatment.(3) Re-hospitalization is sometimes accompanied by unsuccessful treatment, reduced physical, psychological and spiritual well-being. It may also lead to lack of the patients ability to find meaning of life,(4) to be hopefulness and spiritual distress.(5) The results of a study indicated that patients with cancer need supports to overcome fear (57%), hope (58%), meaningful life (50%), and negotiation regarding to dying and death (29%).(1) Review of literatures also shows contradictory findings about the effects of religious and spiritual interventions. The results of some studies have shown positive effects such as; better tolerance of disease,(6) better adherence to therapeutic regimes,(7) improved self-esteem,(8) lower depression and anxiety,(9) and more hope of life(10) following religious or spiritual interventions. On contrast, some studies have reported negative consequences such as anger toward God, anxiety and depression (11) and even thoughts of suicide.^12^

Moreover, patients with cancer want to meet their spiritual needs which may not be necessarily religious needs. Because the meaning and purpose of life is based on a belief system even in people who have no religious beliefs. (13) Studies show that patients increase their demands to meet spiritual needs while facing lethal diseases such as cancer.(14) Assessing spiritual needs and designing interventions based on spiritual needs results in effective adaptation, improved quality of life, and also better interaction with therapeutic plans.(15)

Despite these emphases, the available evidences suggest that holistic cares comprising all aspects of human existence such as physical, mental, social and spiritual aspects has not been considered and especially that patients with cancer had repeatedly reported unmet spiritual needs.(2) Furthermore, spiritual needs of patients with cancer were less considered in oncology wards due to the lack of professional understanding of such needs.(16) Oncologist nurses ought to identify spiritual needs of patients with cancer and meet them through qualified cares.(4) They have golden opportunities to provide spiritual care to patients in need. They can improve patients' spiritual well-being due therapeutic communication with them.(17) Patients with cancer need both physical cares and psychological support to cope with a wide range of challenges from the time of diagnosis to the course of treatment.(18) The main question of the present study was; whether the spiritual based intervention could improve spiritual well-being and hope in patients with cancer who were aware of their disease. Therefore, the present study aimed to examine the impact of spiritual-based intervention on hope and spiritual well-being in patients with cancer.

## Methods

**Diagram 1:**
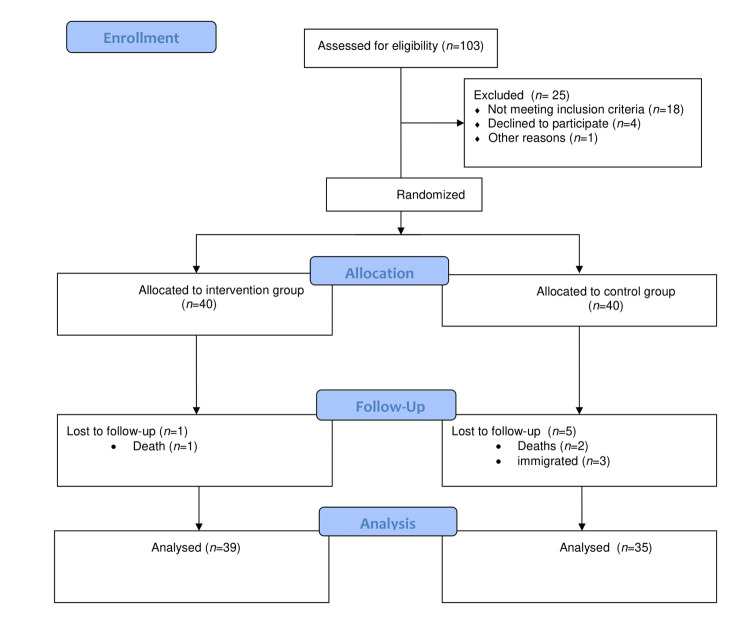
Consolidated Standards of Reporting Trials (CONSORT) of the study

*Design and Participants.* This study is a randomized controlled trial research. The study population was patients with cancer referring to a single chemotherapy ward of Yasuj city, Iran, 2017-2018. One hundred and three patients were assessed for eligibility, however, 80 eligible patients were selected through non-random sampling method and then randomly assigned to one of the two groups of intervention (group A) or control (group B) using block randomization. At first, the groups of intervention and control was labeled with A and B letters, respectively. Next, two blocks namely; AB, BA was created based on the statistical factorial rule (2!: 2×1 =2) since we had two groups in this study. Therefore. We had two participants in each block in which their arrangement differed from each other. We selected blocks from these two blocks using replacement random sampling until the participants of our study were completed. Eighty eligible participants were assigned to one of these two groups (forty participants in each group). However, 74 patients completed this study (4 patients died and 2 patients were reluctant to continue the study). (Diagram 1). The blocked random allocation was designed by the first author, however, participants’ enrollment and assignment to one of the two groups was conducted by the second author of the article.

Inclusion and Exclusion Criteria. Final diagnosis of cancer, undergoing the chemotherapy, range of age: 20-70 years old, patient's awareness of diagnosis, low score of spiritual well-being and hope based on the applied scales and informed consent to participate were considered as the inclusion criteria of this study. Patient’s unwillingness to participate in the study and unmet inclusion criteria were considered to be the exclusion criteria of this study.

Intervention. Spiritual intervention was implemented based on a proposed protocol by Bussing *et al.*(2) in four domains of religious (excellence), existence (meaning and purpose), emotional (relaxation) and social (communication). It was performed over five sessions before starting chemotherapy in the ward (Table 1). The duration of sessions varied from 30 to 50 minutes. The applied strategies in this intervention included interactive negotiation, mutual questioning and answering, short audio or video clips, book introduction, booklet, and expressing personal experience related to the above four domains. Intervention had been performed by the third author of this article who is a nurse with clinical experience working in oncology settings as well as with supporting of a spiritual counselor. The participants in the control group had received usual cares.

**Table 1:** Spiritual based Intervention Protocol

Session	Domain	Main theme	Spirituality-based care
First	Introduction	Patient′s Preparation	Statement of goals and explanation about the intervention
Second	Religious	Excellence	Spiritual resources, relationship with God, Sanctities, Worship
Third	Existence	Meaning and purpose	Meaning of life, Self-Actualization, role function
Fourth	Emotional	Relaxation	Inner calmness, hope, balance, forgiveness, distress, fear
Fifth	Social	Communication	Love and sense of belonging, unity, relationship with spouse, family and friends

Outcome measure. Snyder's Hope Scale, and Paloutzian and Ellison's Spiritual Well-Being Scale (SWBS) were used to collect data. Although the Hope Scale consists of 12 items, however, four items are not included for data analysis due to their deviant nature. Two subscales of factor and strategy (4 questions for each subscale) with an eight- point Likert-type scoring of 1-8 are defined. A score of 1 means completely disagree and score 8 shows completely agree. The global score of hope ranges from 8 to 64. Higher scores represent better levels of hope. The validity and reliability of the Hope Scale were approved in Persian.(19) The SWBS, with a six- point Likert- type scale of 1-6, was used to assess spiritual well-being. It has two subscales of religious and existential well-being (each of which with 10 items). A score of 1 shows completely disagree and score 6 represents completely agree. The scores of each subscale range from 10 to 60. The global score of spiritual well-being ranges from 20 to 120. The scores of spiritual well-being of 20-40 represent low spiritual well-being, scores of 41-99 show moderate spiritual well-being, and scores of 100-120 means high spiritual well-being. The psychometric properties of Persian version of the SWBS were approved.(20) We again checked its reliability using Cronbach’s alpha that it was verified by our study and found a result of α= 0.78.

Data Analysis. Data were collected at a week before intervention as baseline and a week post intervention. The collected data was analyzed using SPSS (Version 21) and through descriptive and inferential statistics such as Chi- square, and Fisher's Exact test for nominal variables. The results of independent samples *t* test and paired samples *t* test were reported for between and within group comparisons, respectively. Since the data distribution of the scores of outcome variables were normal. P values less than 0.05 were statistically considered significant differences.

Ethical Considerations. The informed consent was signed by the participants after explaining purpose of the study. We emphasized the confidentiality of collected data, the voluntary participation and also voluntary withdrawal at each stage of the study. The present study was approved by the Ethics Committee of Yasuj University of Medical Sciences (YUMS) with an ID code; IR.YUMS.REC.1396.137 and the registered number; IRCT20121208011692N2 by website of the Iranian Clinical Trial.

## Results

In the present study, 39 (52.7%) of 74 patients with cancer were in the intervention group and 35 patients (47.3%) were in the control group. The patients had a mean age of 52.9 years (SD=18.1) with (Range; 20-68 years old) (Table 2). The results of the study related to the scale of hope shows that there was no significant difference in mean scores of hope between the two groups in the pre- intervention assessment. However, in the post- intervention, *Independent Samples t test* for between group comparison indicates significant differences (*p*<0.001) in global mean scores of hope and also subscales of factor and strategy for the patients in the intervention group compared with the patients in the control group (Table 3). In addition, mean differences for global scores of hope (13.7), and sub-scales of factor (6.6) and strategy (7.2) are observed for the patients in the intervention group. These mean differences were statistically significant based on the results of *Paired Samples t test* (*p*<0.001).

The results of the study related to the scale of spiritual well-being shows that there were no significant differences in mean scores of spiritual well-being between the two groups in the pre-intervention assessment. On contrast, our findings indicate significant differences for global mean scores of spiritual well-being and subscales of existence well-being, and religious well-being following spiritual intervention compared with the control group (table 4). The results of *Paired Samples t test* in within group comparison, presents statistical mean difference for global spiritual well-being (21.7), subscales of existential well-being (12.4) and religious well-being (9.2) for the patients in the intervention group, but no significant mean differences are observed for the patients in the control group.

**Table 2:** Participants’ demographic characteristic by groups

Group Variables	Group Variables	Intervention	Control	Total	*p-value*
Group Variables	Group Variables	*n*=39	*n*=35	*n*=74	*p-value*
Age: Mean±SD	Age: Mean±SD	20.4 ±51.9	16.1 ±53.9	18.1±52.9	0.6
Duration of cancer diagnosis: Mean±SD	Duration of cancer diagnosis: Mean±SD	14.1±15.2	10.6±16.4	12.5±15.8	0.6
Duration of chemotherapy: Mean±SD	Duration of chemotherapy: Mean±SD	6.1±7.5	5.1±6.7	5.5±7.1	0.7
Sex: n(%)	Male	25 (64.1)	17 (48.6)	42 (56.7)	0.2
Sex: n(%)	Female	14 (35.9)	18 (56.3)	32(43.3)	0.2
Marital status	Single	2 (5.1)	6 (17.1)	8 (10.8)	0.09
n (%)	Married	37 (94.9)	29 (82.9)	66(89.2)	0.09
Education	Primary	26 (66.7)	26 (74.3)	52 (70.4)	0.09
n (%)	Secondary school	4 (10.3)	7 (20)	11 (14.8)	0.08
Education	Diploma and higher	9 (23.1)	2 (5.7)	11(14.8 )	0.08

**Table 3:** Mean scores of hope in both the intervention and control groups

Group	Group	Intervention	Control	*Independent Samples t test*	*Independent Samples t test*
Dimension / Time	Dimension / Time	Mean± SD	Mean± SD	Mean difference	*p-value*
Factor	Pre	3.1±23.7	2.21±20	3.7	0.1
Factor	Post	1.34±30.3	2.4±19.8	10.5	0.001
Strategy	Pre	3.2 ±23.5	2.1±21.7	1.8	0.2
Strategy	Post	1.3±30.7	1.7±19.9	10.8	0.001
Global Hope	Pre	5.1±47.2	3.7 ±44.4	2.8	0.06
Global Hope	Post	2.1 ±60.9	3.3±39.8	21.1	0.001

**Table 4:** Mean scores of spiritual well-being in both the intervention and control groups

Group	Group	Intervention	Control	*Independent Samples t test*	*Independent Samples t test*
Dimension / Time	Dimension / Time	Mean± SD	Mean± SD	Mean difference	*p-value*
Religion health	Pre	39.8±9	36.4±2.6	3.4	0.06
Religion health	Post	49±1.3	36.7±2.3	12.3	0.001
Existential health	Pre	32.9±7	34.7±2.3	1.8	0.3
Existential health	Post	45.3±3.7	34.9±2.3	10.4	0.001
Global Spiritual Well-being	Pre	72.6±6.3	70.9±3.1	1.7	0.05
Global Spiritual Well-being	Post	94.3±4.7	71.6±2.9	22.7	0.001

## Discussion

According to the question of this study, the findings indicated that the spiritual- intervention improved spiritual well-being and hope in patients with cancer undergoing the chemotherapy. In other words, respond to spiritual needs led to positive changes in spiritual well-being and hopefulness.(21) The findings of this study is similar to published studies which have indicated benefits of social support,(22) quality of life,(23) patient’s recovery,^7^ and strengthened and facilitated interpersonal communication,(24) reduced symptoms and frustration^25^ following spiritual or religious interventions. Finding of a qualitative survey by Zumstein-Shaha and colleagues showed that patients with cancer in struggling with disease often use religion/spirituality and rituals to find meaning.(26) Another correlational study has showed that cancer patients undergoing chemotherapy who had a high religious/spiritual coping score were found to have a higher level of hope.(27) The results of a study by Mansurifard and colleagues indicated spiritual health of adolescents with cancer was promoted following spiritual cares,(28) which is in line with our study. However, the findings of our study is not similar to a study by Kang and colleagues, in which meaning of life of adolescents with advanced cancer had been improved following logo therapy, however, no significant changes were observed for spiritual well-being in both the intervention and control groups.(29) A study by Delavari and Nasirian showed improved mental health and reduced anxiety in mothers of children with cancer following logo therapy.(30) On contrast, failure to provide spritual care is associated with spiritual distress, then increased healthcare costs, risk of depression and anxiety(31) which are important challenges to meet spiritual needs of patients with cancer.(32)

Despite reporting similar results in the mentioned studies, they also have methodological limitations that should be considered when comparing their results. Providing spiritual cares to patients with cancer is an interdisciplinary work including oncologists, oncology nurses, chaplains, psychologists and even patients with cancer and families. There is a fact that both patients with cancer search spiritual support such as hope, meaning, spiritual well-being interdisciplinary team agree that spiritual supports promote spiritual health of patients in oncology settings.(33) Assessing spiritual needs and recognize spiritual distresses of patients with cancer are key elements of holistic care.(34) Patients with cancer may experience spiritual distress due to uncertainty regarding prognosis and deteriorating health, cancer recurrence.(35) Indicators of hope and spiritual well-being are important in this regard.(36) Moreo er, spirituality is considered as an important predictor of emotional, functional, social well-being and quality of life of patients and families with cancer.(37)

Strength and Limitations. Randomized allocation is strength of this study. However, the current study has some limitations that caution in needed when generalizing its results. First, the current study was conducted in a single chemotherapy ward in which all participated patients were Muslims with same belief system. Thus, the participants did not have a diverse religious profile. Belief system may be used as strategy to cope with life-threatening diseases like cancer. Second, spirituality is a multidimensional and absolutely individual concept,(38) which may be associated with religion. (2) Religious people exhibit less spiritual distress due to higher psychosocial adaptation.(39) Patients with cancer may rely on religious issues as important adaptive resources due to the lethal nature of cancer.(40) The results of some studies have shown that patients' spiritual needs vary based on their religious beliefs; and patients without religious beliefs had lower levels of hope and well-being than patients with religious beliefs(41,42) Thus, future studies with designing different religious affiliations and ethnicity are suggested to better clinical judgment regarding the impacts of spiritual based interventions. More investigations eliciting patients' responses can help to better understand influence of spirituality and religious on patients and their needs throughout the trajectory of a cancer diagnosis, treatment, and transition to end of life. In this ways, Spirituality interventions will be supported in clinical practice by evidence based nursing.

## Conclusion

The present study indicated that the spiritual-based intervention could improve the spiritual well-being and hope in patients with cancer. The importance of providing spiritual interventions to meet cancer patients’ spiritual needs such as hope and spiritual well‑being is again highlighted by this study. Spiritual interventions as an important component of holistic care should be incorporated into the plan of nursing cares for both patients with cancer and families. Indicators such as spiritual well-being and hope are helpful to assess the effectiveness of these types of interventions in patients with cancer. Our study was a small research in a single chemotherapy, however, further investigations is needed in this area on cancer survivors, patients at the end of life as well as caregivers. Moreover, further research with different settings or the study populations with different sociocultural contexts may be useful to understand how spirituality affect patient to cope with cancer from at the point of diagnosis, treatment, disease progression and even facing with his/her own mortality.
